# A multicenter retrospective analysis of canine idiopathic epilepsy in China

**DOI:** 10.3389/fvets.2026.1808718

**Published:** 2026-06-02

**Authors:** Xing-Wang Zhang, Yu-Cheng Guan, Zhi-Jiang Liu, Ling-Sen Lou, Zhi-Hao Liu, Andrea Tipold, Yu-Wei Lin

**Affiliations:** 1 Tianhong Pet Hospital, New Ruipeng Pet Healthcare Group, Hefei, Anhui, China; 2 Ainuo Blessing Veterinary Hospital, New Ruipeng Pet Healthcare Group, Guangzhou, Guangdong, China; 3 Zhengzhou Pet Health Center Hospital, New Ruipeng Pet Healthcare Group, Zhengzhou, Henan, China; 4 Hangzhou Meilian Zhonghe Animal Hospital, New Ruipeng Pet Healthcare Group, Hangzhou, Zhejiang, China; 5 Qingdao Ainuo Central Hospital, New Ruipeng Pet Healthcare Group, Qingdao, Shandong, China; 6 Department of Small Animal Medicine and Surgery, University of Veterinary Medicine, Hannover, Germany; 7 Naughty Family Animal Hospital, New Ruipeng Pet Healthcare Group, Shanghai, China

**Keywords:** canine, idiopathic epilepsy, multicenter study, predisposing factor, retrospective analysis, seizure

## Abstract

**Objective:**

Canine idiopathic epilepsy (IE) is one of the most common neurological diseases in veterinary medicine, with no comprehensive study in China. This study collected IE cases from five large referral pet hospitals and conducted a multicenter retrospective analysis in order to supplement the clinical data of canine IE in China, clarify the predisposing factors, diagnostic and therapeutic characteristics of canine IE in the China, and provide regional clinical data for cross-country comparison of canine epilepsy diagnosis and treatment.

**Methods:**

Canine patients diagnosed with epilepsy from five pet hospitals in China between 2019 and 2023 were collected. IE cases were diagnosed based on the consensus of the International Veterinary Epilepsy Task Force (IVETF). Breed, age, body condition score (BCS), diagnosis, treatment and follow-up were included for analysis.

**Results:**

A total of 211 cases with IE were included in the study. Male dogs (*p* < 0.05), small dogs weighing <10 kg (*p* < 0.05), poodles (*p* < 0.05) had a significantly higher risk to be diagnosed with IE. The age of first seizure episode was mainly between 1 and 5 years, with an average of 4 years. Most patients had seizure episodes with a mean duration of less than 2 min (78.9%). Generalized seizures with tonic–clonic convulsions, accompanied by autonomic signs, were the most frequently observed type. The incidence of cluster seizures (CS) was 38.9%, while status epilepticus (SE) occurred in 12.4% of cases. Phenobarbital was administered in 89.1% of cases, with 58.3% receiving monotherapy with phenobarbital; 74.8% of these cases achieved good seizure control (seizure-free or a clinically meaningful reduction in seizure frequency). The disease-related mortality rate in these cases was approximately 1%.

**Conclusion:**

This study reveals a significant diagnostic gap in China, with 18.7% of cases exceeding a one-year delay to diagnosis, likely due to a shortage of specialists and owner-related factors. Optimizing clinical management should prioritize reducing this delay through enhanced practitioner and owner education, while reinforcing Phenobarbital as a highly effective (93.8% success rate) first-line monotherapy. Addressing these barriers could facilitate earlier intervention and improve therapeutic outcomes for dogs with idiopathic epilepsy in this region.

## 1 Introduction

**Figure 1:**
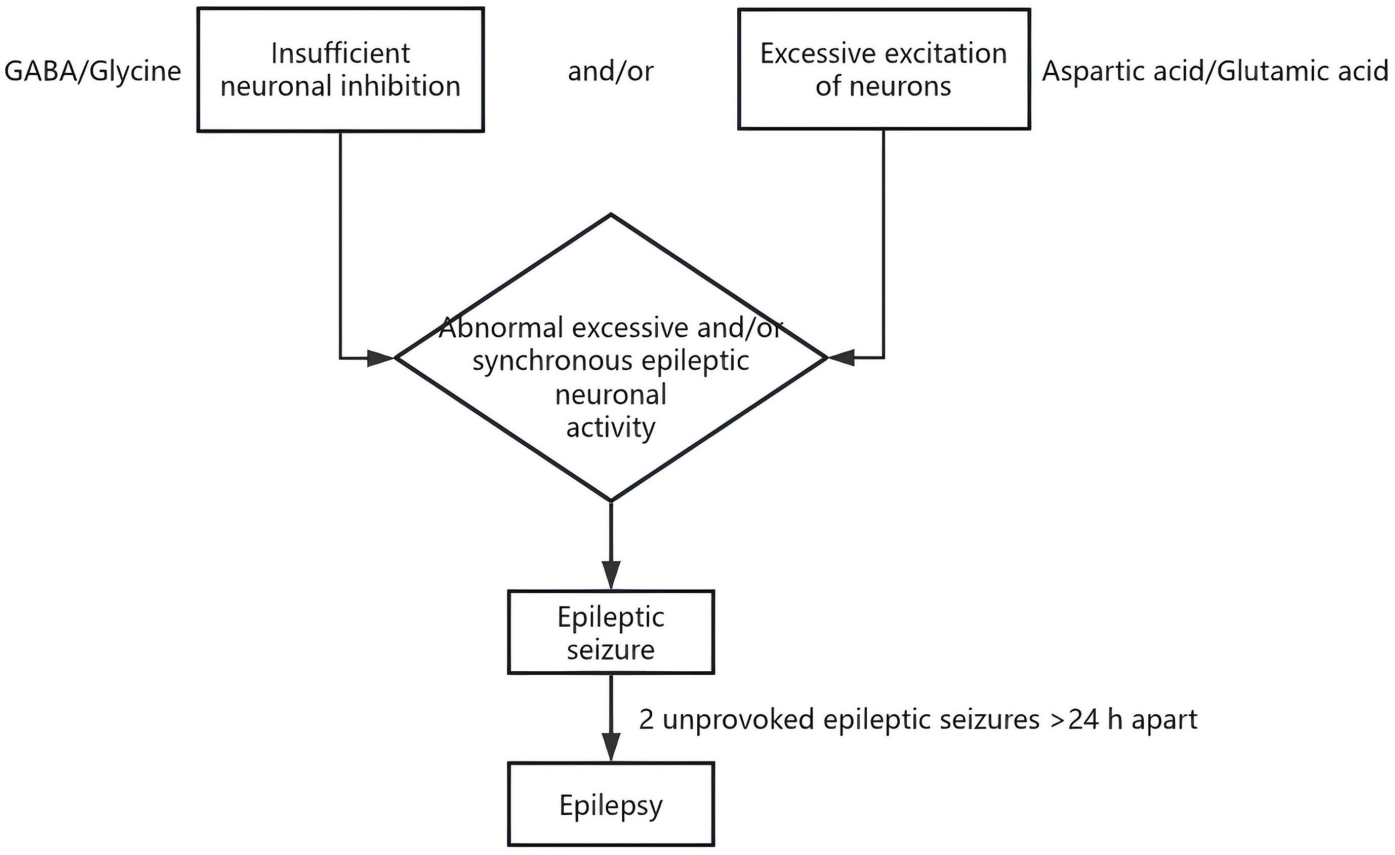
Speculation on pathogenesis of canine epilepsy.

Epilepsy is a neurological disorder characterized by recurrent seizures over a long period. Epileptic seizures are characterized by a transient occurrence of clinical signs due to excessive or overly synchronous neuronal activity in the brain. The exact mechanism of its occurrence is not yet fully understood, but the main possible causes include insufficient neuronal inhibition, excessive neuronal excitation, or a combination of both (1, 2), as shown in Figure 1. Epilepsy is usually closely associated with specific clinical signs, which have a direct or potential impact on the quality of life of animals and their owners (3). Most dogs with idiopathic epilepsy (IE) require lifelong treatment or management (3). The diagnosis of epilepsy needs to be distinguished from syncope, narcolepsy, myasthenia gravis, peripheral vestibular dysfunction, and other abnormal movements or behaviors (4, 5). Paroxysmal dyskinesia (PD) represents a major differential diagnosis. While Thomas provided a detailed description and differentiation of non-epileptic paroxysmal diseases in 2010 (5), the International Veterinary Canine Dyskinesia Task Force has recently published a consensus statement establishing clear terminology and classification for PD (6). In clinics, it is somewhat challenging to distinguish between epileptic seizures and non-epileptic paroxysmal attacks. Thomas WB provided 2010 a detailed description and differentiation of epilepsy and various types of paroxysmal non-epileptic diseases in his research (5). However, there remains a lack of unified consensus on the diagnosis of IE. Therefore, IVETF proposed a three-level confidence level diagnostic approach (TIER 1–3) for IE. A higher confidence level results in a higher credibility of the diagnosis of IE (7). Previous research has shown that the typical age of onset for canine IE is 6 months to 6 years (7). Patients who experienced their first seizure before the age of 6 months can suffer from IE, but congenital abnormalities and metabolic disorders must be ruled out (5). Approximately 35% patients whose age at onset is over 5 years are diagnosed with IE (8). Canine IE exhibits a distinct breed predisposition. Susceptible breeds have been reported including Golden Retrievers (9), Labrador Retrievers (10), Standard Poodles (11), Border Collies (12), and Bernese Mountain Dogs (13). The epidemiological landscape of canine idiopathic epilepsy (IE) is known to vary by region, largely influenced by local breed popularity and socioeconomic factors. While detailed studies have emerged from the United Kingdom (14, 15), Sweden (16), Germany (17) and Japan (18), providing critical insights into breed predispositions and regional outcomes, no such comprehensive data exists for the rapidly expanding pet population in China. This creates a significant gap in the global understanding of canine IE. Therefore, the purpose of this study was to conduct a multicenter retrospective analysis to characterize the signalment, diagnostic features, and treatment outcomes of canine IE in China, thereby supplementing the international database with regional findings.

## 2 Materials and methods

### 2.1 Animals

Epilepsy cases from five hospitals within the New Ruipeng Pet Healthcare Group—Tianhong (Hefei), Ainuo Blessing (Guangzhou), Zhengzhou Pet Health Center (Zhengzhou), Meilian Zhonghe (Hangzhou), and Ainuo Center (Qingdao)—were retrospectively collected between 2019 and 2023. A unified Zilong Hospital Information System (HIS) is employed across all these facilities, allowing for a centralized search. Potential cases were identified by querying the Zilong database using the search terms “epilepsy” and “seizure” to retrieve relevant medical records. Subsequently, cases that met the inclusion criteria were statistically analyzed. The inclusion criteria are based on the consensus proposal on diagnosis of epilepsy in dogs by the International Veterinary Epilepsy Task Force published in 2015 (7), as shown in Table 1.

**Table 1:** Criteria for the diagnosis of IE, as published by the International Veterinary Epilepsy Task Force consensus proposal: diagnostic approach to epilepsy in dogs (5).

Confidence level	Definition
Tier I	≥2 unprovoked epileptic seizures occurring at least 24 h apart Age at epileptic seizure onset of between 6 months and 6 years Unremarkable inter-ictal physical and neurological examination no significant abnormalities on minimum data base blood tests and urinalysis*
Tier II	In addition to factors listed in tier I Unremarkable fasting and post-prandial bile acids Magnetic resonance imaging (MRI) of the brain (based on an epilepsy-specific brain MRI protocol) and cerebrospinal fluid (CSF) analysis*
Tier III	In addition to factors listed in tier I and II Electroencephalographic (ECG)* abnormalities characteristic for seizure disorders.

*Urine analysis, CSF and ECG is not essentially required in this study.

Following IVETF guidelines, idiopathic epilepsy (IE) was diagnosed in patients meeting at least Tier I confidence level criteria. Additionally, dogs exhibiting interictal neurological deficits or brain MRI abnormalities were included only if these findings were strictly temporary or treatment-related:

1. Transient MRI signal alterations: patients with focal T2W/FLAIR hyperintensities and T1W hypointensities restricted to the piriform or temporal lobes were included, provided these changes occurred within 14 days of a seizure and were interpreted as seizure-induced brain edema (postictal changes) rather than structural lesions (19).
2. Reversible neurological deficits: cases with neurological abnormalities were included only when such deficits were conclusively attributed to the side effects of anti-seizure drugs (ASDs) or were identified as transient postictal phenomena (7), with a return to normal baseline confirmed during subsequent clinical follow-ups.

Transient postictal lesions were differentiated from structural epilepsy based on their characteristic anatomical location (piriform/temporal lobes) and lack of progressive deficits in follow-up exams.

In this cohort, 160 dogs (75.8%) met the IVETF Tier II criteria by undergoing brain MRI. The remaining cases were diagnosed at Tier I level. CSF analysis was recommended for all Tier II candidates but was frequently declined by owners due to financial constraints or concerns regarding the invasiveness of the procedure; consequently, CSF data were not included in the formal statistical analysis. In cases with MRI abnormalities interpreted as postictal changes, lesions were predominantly localized to the piriform and temporal lobes; no other structural abnormalities suggestive of structural epilepsy were identified.

The protocol for this multicenter retrospective study was structured to comply with local ethical guidelines and Chinese data protection regulations. As the study involved the analysis of pre-existing, anonymized clinical data from hospital information systems (HIS) and did not involve any prospective intervention or deviation from standard clinical care, formal ethical approval was not necessary according to local regulations. All data were handled anonymously to ensure patient and owner confidentiality.

### 2.2 Procedures

For the included cases, signalment (age at first seizure event, breed, gender, body condition score (BCS), neutering status), duration of seizures, seizure types (generalized/focal/autonomic signs), occurrence of status epilepticus (SE) or cluster seizures (CS), and treatment were recorded. We conducted statistical analyses on each parameter separately, summarizing the characteristics of IE in dogs, management plans, and treatment outcomes. Seizure freedom is the primary goal of the therapeutic management of canine and feline epilepsy patients (3). Partial therapeutic success includes reduction of seizure frequency including information on seizure incidence, seizure severity, or reduction in frequency of CS and SE (3). To classify different outcomes of therapeutic interventions, we defined “seizure free,” a seizure frequency of less than one seizure every 3 months (seizure reduction) and reduction of seizure density, severity and the frequency of CS and SE as the outcome “well controlled.”

**Figure 2:**
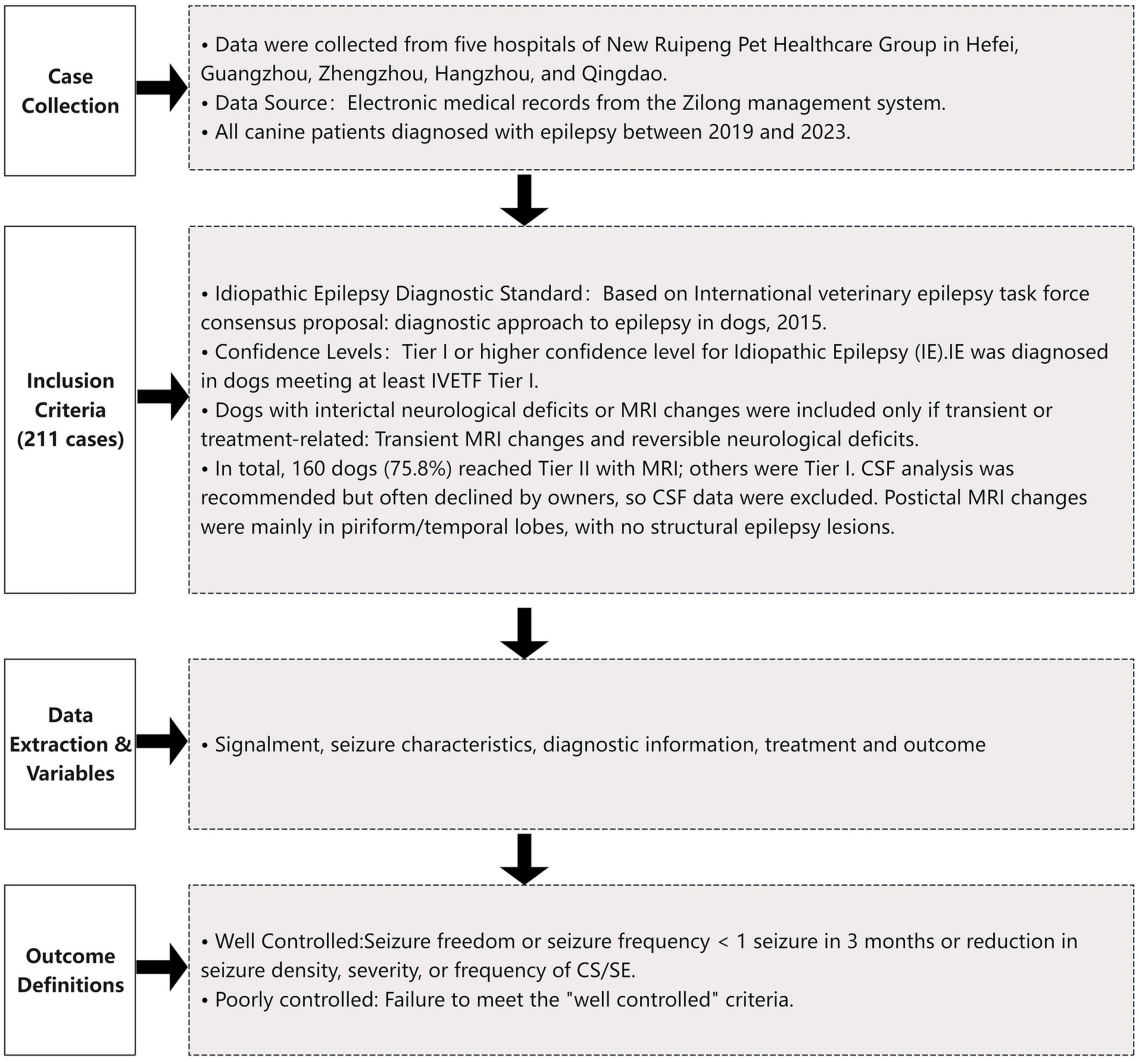
Multicenter retrospective study of canine idiopathic epilepsy in China: overview.

**Figure 3:**
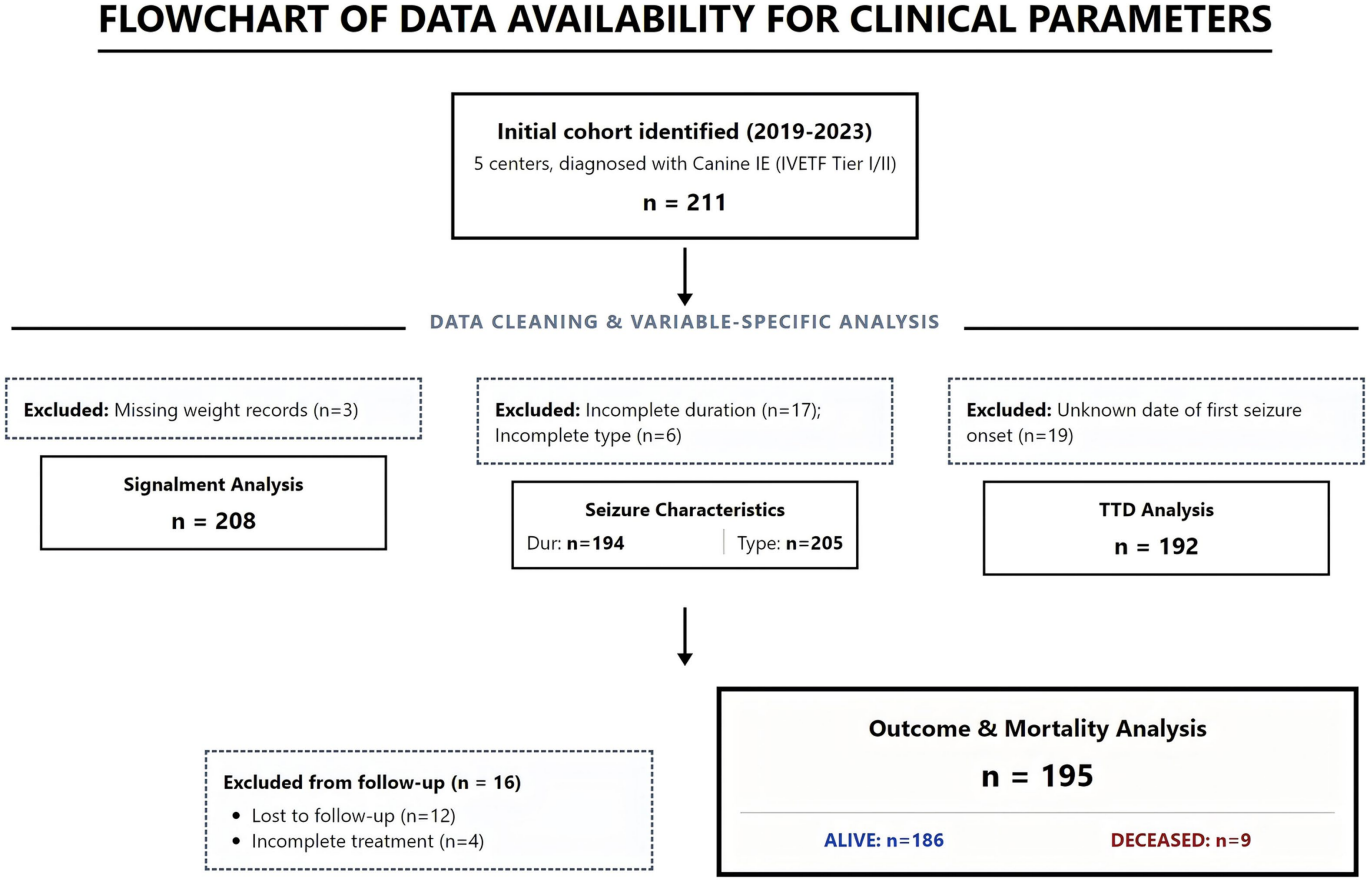
Flowchart of the case selection process and data distribution for sub-analyses. The diagram illustrates the refinement from the initial multicenter cohort (*n* = 211) to the specific populations analyzed for signalment, seizure characteristics, time to diagnosis (TTD), and long-term treatment outcomes.

The overall patient selection and data refinement process are summarized in Figure 2. Due to the retrospective nature of this multicenter study, the specific number of cases included in each sub-analysis (e.g., body weight, seizure duration, and treatment outcome) was determined by the availability of complete medical records within the hospital information system (HIS). Data attrition for specific parameters occurred primarily due to missing data fields or loss to follow-up, and the exact denominators used for each clinical evaluation are detailed in Figure 3.

### 2.3 Statistical analysis

Statistical analysis of the obtained data was performed using IBM SPSS Statistics 26.0 software, and the chi-square test of independence was used for intergroup difference testing. A *p*-value <0.05 was considered statistically significant.

## 3 Results

### 3.1 Animals with IE

This study involved a total of 211 dogs with IE from five pet hospitals in five regions in China from 2019 to 2023. Fifty-one cases (24.2%) were diagnosed at Confidence Level I, and 160 cases (75.8%) met the criteria for Confidence Level II. On the owner’s request, urine sampling and cerebrospinal fluid analysis was not performed in some cases. No cases fully met the diagnostic criteria for confidence level III (additional evaluation of electroencephalogram). Over the described 5 year period, 52,926 canine patients were presented in the five hospitals. Of these cases 559 were diagnosed with epilepsy, which included 211 dogs with IE. The incidence of epilepsy in this hospital population was 1.1%, the incidence of IE was 0.4%, and IE accounted for 37.7% of all epilepsy cases.

### 3.2 Signalment

Of the 211 dogs with IE, 10 had missing gender records and were therefore excluded from statistical analysis. Dogs with IE included 25.9% (52/201) neutered dogs and 74.1% (149/201) intact dogs, with 48.3% (97/201) intact males, 25.9% (52/201) intact females, 16.9% (34/201) neutered male dogs and 8.9% (18/201) neutered female dogs.

Dogs with a body weight of 0–5 kg and 5–10 kg accounted for 24.0% (50/208) and 35.6% (74/208) of cases, respectively (Table 2). The average weight was 12.7 kg. The mean age at first seizure was 48.8 months. Sixty-nine percent (140/203) of patients were 12–60 months of age. The mean time to diagnosis (TTD) from the initial seizure onset was 8.2 months (range: 0 days to 48 months, *n* = 192). In this cohort, 81.3% of dogs (*n* = 156/192) were diagnosed within 1 year of the first seizure event, while 18.7% (*n* = 36/192) experienced a diagnostic delay exceeding 12 months.

**Table 2:** Body weight distribution in 208 dogs with idiopathic epilepsy.

Body weight/kg	0 ~ 5	5 ~ 10	10 ~ 15	15 ~ 20	20 ~ 25	25 ~ 30	30 ~ 35	35 ~ 40	40 ~ 45	45 ~ 50	50 ~ 55
Number of dogs	50	74	21	11	14	14	14	4	3	2	1
Percentage %	24.0	35.6	10.1	5.3	6.7	6.7	6.7	2.0	1.4	1.0	0.5

Body condition was assessed using a 9-point scale (20), categorized as underweight (BCS 1–3 of 9), ideal (BCS 4–5 of 9), or overweight (BCS 6–9 of 9), consistent with the WSAVA nutritional assessment guidelines (21). In our study, 67.0% (132/197) dogs were classified as ideal, 27.9% (55/197) as overweight, and 5.1% (10/197) as underweight. A total of 28 breeds were presented. Poodles were the most frequently dogs diagnosed with IE and included 26.7% (54/202). 11.4% (23/202) were mixed-breed dogs and 9.9% (20/202) were Bichon Frise (Table 3).

**Table 3:** Breed distribution in 202 dogs with idiopathic epilepsy.

Breed	Number of dogs	Percentage
Poodle	54	26.7%
Mixed breed dog	23	11.4%
Bichon Frise	20	9.9%
Golden Retriever	15	7.4%
Siberian Husky	11	5.4%
Miniature Schnauzers	9	4.5%
French bulldog	9	4.5%
Pomeranian	8	4.0%
Welsh corgi	8	4.0%
Alaskan Malamute	6	3.0%
Labrador Retriever	6	3.0%
Border Collie	5	2.5%
Samoyed	4	2.0%
Yorkshire Terrier	3	1.5%
West Highland White Terrier	3	1.5%
Akita	3	1.5%
Shetland Sheepdog	2	1.0%
Dalmatian	2	1.0%
Others	11	5.4%

### 3.3 Seizure characteristics

Seizure duration was categorized into three intervals: ≤2 min, 2–5 min, and >5 min. 78.9% (153/194) of cases had seizures with a duration of ≤2 min, 17.0% (33/194) from 2 to 5 min and 4.1% (8/194) of over 5 min (Table 4). The proportion of cases with a seizure duration of less than 2 min was significantly higher than those with a duration of 2 to 5 min and more than 5 min (*p* < 0.05).

**Table 4:** Seizure characteristics in 211 dogs with idiopathic epilepsy in China.

Seizure characteristics absolute number of dogs (percentage)				
Duration of the ictus (*n* = 194)	<2 min	2–5 min		>5 min
	153 (78.9%)	33 (17.0%)		8 (4.1%)
Type of seizures (*n* = 205)	Focal only	Generalized only		Combined
	10 (4.9%)	166 (81.0%)		29 (14.1%)
Emergency seizure episode	Status epilepticus only	Cluster seizures only	Both	Not observed
	6 (2.8%)	62 (29.4%)	18 (8.5%)	125 (59.3%)
Autonomic signs (*n* = 201)	103 (51.2%)			

Generalized seizures were the most frequently observed seizure type, accounting for 94.7% (195/205) of cases. Focal seizures were observed in 39 cases (19.4%; 195/205). Among them, 10 cases (4.9%) only had focal seizures. In 29 cases both generalized and focal seizures occurred.

There were 86 dogs with emergency seizure episodes: 2.8% (6/211) of dogs with SE occurred, 29.4% (62/211) experienced CS and 8.5% cases (18/211) had both. Autonomic signs during seizures (such as vomiting, urination, defecation, etc.) were recorded in 51.2% (103/201) of the cases with detailed information shown in Table 4.

### 3.4 Long term treatment and outcomes

The administration and outcomes of long-term ASDs in 195 dogs with IE were summarized (as detailed in Table 5). Phenobarbital (PB) was the most commonly administered ASD. A total of 123 dogs received monotherapy, 121 of which were treated with PB as the sole agent. Of the 96 dogs treated with PB monotherapy with available follow-up data, 90 (93.8%) achieved well control. Two dogs received gabapentin (GBP) monotherapy.

**Table 5:** Long term administration of antiseizure drugs (ASDs) in 195 dogs with idiopathic epilepsy and outcome.

Regimen	Number of dogs	Ratio of well-controlled to dogs with follow up examinations	Number of deaths
	*N* = 195		*N* = 9
One single drug	*n* = 123		
PB	121	90/96 (93.8%)	5
GBP	2	/	/
Two-drug combination	*n* = 54		
PB + KBr	4	1/3 (33.3%)	/
PB + LEV	39	33/35 (94.3%)	/
PB + ZNS	2	2/2 (100%)	/
PB + GBP	7	3/4 (75%)	/
PB + IMP	1	1/1 (100%)	/
LEV+GBP	1	1/1 (100%)	/
Three-or-more -drug combination	*n* = 13		
PB + LEV+GBP	9	5/7 (71.4%)	1
PB + LEV+PGB	3	3/3 (100%)	/
PB + LEV+IMP	1	0/1 (0%)	/
No long-term anticonvulsants	4	/	3

PB, Phenobarbital; GBP, gabapentin; KBr, potassium bromide; LEV, levetiracetam; ZNS, zonisamide; IMP, imepitoin.

Fifty-four dogs received combination therapy with two ASDs. The most common combination was PB plus levetiracetam (LEV) (*n* = 39), with 33 of 35 (94.3%) achieving good control. Other two-drug regimens included PB plus potassium bromide (KBr) (*n* = 4), PB plus zonisamide (ZNS) (*n* = 2), PB plus GBP (*n* = 7), PB plus imepitoin (IMP) (*n* = 1), and LEV plus GBP (*n* = 1).

Thirteen dogs received a combination of three or more drugs. The most frequent triple therapy was PB plus LEV plus GBP (*n* = 9), with 5 of 7 (71.4%) well-controlled. Other regimens included PB plus LEV plus pregabalin (PGB) (*n* = 3) and PB plus LEV plus IMP (*n* = 1).

### 3.5 Mortality

Of the 195 dogs with long-term follow-up, 9 deaths (4.6%) were recorded. Five deaths (2.6%) were directly attributed to epilepsy, including two cases that died during status epilepticus (SE) and three cases that underwent euthanasia due to poor quality of life (QoL) secondary to refractory seizures. The remaining four deaths were attributed to unrelated comorbidities, including suspected toxicosis, diabetes mellitus, renal failure, and gastrointestinal disease. At the time of writing this manuscript, 186 dogs (95.4%) were still alive.

## 4 Discussion

According to the IVETF consensus statement (7), epilepsy is defined as a brain disease characterized by an enduring predisposition to generate epileptic seizures. The IVETF has classified canine epilepsy into three etiological categories: IE, structural epilepsy, and epilepsy of unknown origin (22). Canine IE includes dogs displaying seizures with (1) a clearly identified epilepsy-causing gene; (2) breeds/families with a high incidence rate and genetic risk; and (3) an unknown cause of epilepsy but no structural lesions (22). Pathological mechanism of IE is still not completely uncovered. Although there are many research articles and reports about epilepsy in veterinary medicine, there has been no comprehensive retrospective study of canine IE in China, which could be compared to data of other countries. In terms of prevalence of epilepsy, several studies showed an incidence around 0.75 to 1.87% (15, 18). In our hospital population, 1.1% (559/52926) of cases were diagnosed with epilepsy in 5 hospitals in China within 5 years (from 2019 to 2023). The described population has a similar incidence rate of epilepsy as found in dog populations in other studies.

In the current study 211 cases with IE were described, with an incidence rate of approximately 0.4%. These are the first published data in China associated with canine epilepsy and IE, which are slightly lower than the rate of 0.9% published by Hamamoto et al. (18). IE accounted for 37.7% of epilepsy cases in our study, while 25–54.9% were described in other studies (23–26). Of the 211 dogs, 51 cases met Confidence Level I, accounting for 24.2%, and 160 cases met Confidence Level II, accounting for 75.8%. The diagnostic process for Confidence Level II and above can more accurately diagnose canine IE.

Our study revealed that intact (*p* < 0.05) and male dogs were more frequently affected by IE, which is consistent with the results of Monteiro et al. (20). Small dogs suffering from IE were significantly more frequently observed than large breed dogs (*p* < 0.05) and Poodles had a significantly higher incidence rate than other breeds (*p* < 0.05), followed by mixed breed dogs, Bichon Frise and Golden Retrievers. However, these findings may not solely reflect an inherent breed predisposition, but may also be strongly associated with the regional popularity of these breeds as companion animals in China. Our cohort exhibits a breed distribution that closely aligns with the general canine population in China. According to 2024 Apa-institute data (27), small breeds (<9 kg) comprise 54.7% of the national pet dog population, with Poodles (18.1%) and Bichon Frise (9.5%) being the most prevalent (21). The high representation of these breeds in our study likely reflects regional ownership trends rather than a unique regional breed predisposition.

Therefore, although the data show that the number of Poodles with IE is significantly higher than other breeds, it does not objectively indicate that Poodles have the highest incidence of epilepsy worldwide. It may only reflect the situation in China. Average age at onset is 48.8 months. Onset age is mainly between 1 year and 5 years, which is consistent with the research results of Jaggy et al. (28).

The clinical landscape of IE in China is characterized by a rapid expansion of diagnostic infrastructure juxtaposed with an evolving specialized workforce. In our cohort, the mean time to diagnosis (TTD) was 8.2 months (range: 0 days to 48 months, *n* = 192), with 18.7% of cases exceeding a 12-month operational threshold defined as “prolonged diagnostic delay.” While seizure frequency is a traditional metric of severity, this 1-year temporal cutoff is particularly relevant in the regional context. It reflecting a notable interval between the first observed seizure and definitive specialist diagnosis. The underlying drivers for this delay require cautious interpretation. Potential hypotheses include initial intermittent seizure frequency that may not initially alarm owners, or the logistical challenges of reaching specialized neurology services. Future research utilizing owner and practitioner surveys would be required to objectively assess the socio-professional factors influencing these diagnostic timelines. Furthermore, while 75% of the owners in this cohort consented to MRI under general anesthesia, diagnostic pathways for Tier II confirmation remained incomplete in many cases due to the frequent refusal of CSF analysis. This suggests that owner decision-making may be influenced more by the perceived risks of specific invasive procedures (e.g., cisterna magnum sampling) than by the risks of anesthesia itself. Clinically, it has been observed that owners often harbor greater apprehension toward neuro-invasive diagnostics compared to non-invasive imaging. However, as this study did not include a structured survey of owner motivations, this remains a clinical hypothesis. Future prospective research is necessary to objectively assess the socio-cultural factors and risk perceptions that influence diagnostic compliance in the Chinese canine population. Notably, nearly 80% of our cases exhibited a mean seizure duration of less than 2 min, aligning with typical isolated epileptic events. While our data highlights a significant subset with prolonged TTD, further prospective studies are warranted to determine whether earlier diagnostic intervention and reduced TTD directly correlate with improved long-term seizure control and prognosis in the Chinese canine population.

Obesity is recognized as a significant comorbidity in human epilepsy and has been potentially linked to drug resistance (29). However, the relationship between body condition and seizure outcome was not statistically evaluated in this study. Further prospective studies are warranted to investigate whether metabolic status influences ASD efficacy in canine IE.

In the statistical analysis of seizure types, 94.7% had definitively experienced generalized seizures, and 81.0% had only experienced generalized seizures without any observed focal seizures. 19.0% of the cases had definitively experienced focal seizures, and 4.9% had only experienced focal seizures. There were a total of 29 cases that had experienced both types of seizures. The results of our group of studies are consistent with those obtained by Armaşu M et al. (30). This indicates that canine IE clinically most commonly presents as generalized seizures or that patients with seizures are only referred to pet hospitals with generalized seizures, but some cases may be accompanied by or have previously experienced focal seizures.

In our cohort, the prevalence of cluster seizures (CS) was significantly higher than that of status epilepticus (SE), which is consistent with findings by Hamamoto et al. (18). Autonomic signs were recorded in 51.2% of cases (*n* = 103/201). However, these figures likely represent a conservative estimate. Given the retrospective nature of this study, subtle autonomic signs or brief clusters seizures might have been under-reported by owners or not fully captured in the electronic medical records, particularly in cases with lower seizure frequency.

Long-term treatment outcome and mortality were evaluated in 195 dogs. Of the initial 211 cases, 16 were excluded from this specific analysis: 12 dogs were lost to follow-up after the first visit, and 4 dogs had incomplete treatment records that precluded accurate assessment (Figure 2). Regarding IE treatment, ASDs were not widely available for veterinary use in China in the early stages of clinical practice. The majority of dogs (123/195, 63.1%) were managed with a single antiseizure drug. Within this monotherapy group, PB was clearly the first-choice treatment, used in 121 of the 123 cases (98.4% of monotherapies). This underscores PB’s role as the cornerstone of initial medical management for canine IE in this study population. According to the American Veterinary Internal Medicine College (ACVIM) consensus statement on canine epilepsy management (31), phenobarbital is recommended as a first-line antiseizure drug, while levetiracetam lacks relevant data for use as a first-line drug. Previous studies (32–34) revealed similar results concerning seizure control effects as shown in our study.

Two-Drug combinations also showed favorable outcomes in most regimens. The most common combination, PB plus LEV, resulted in 33 out of 35 dogs (94.3%) being well-controlled. Other PB-based combinations also reported good control rates (75–100%), although sample sizes for individual combinations were small. Three-or-More-Drug combinations were reserved for more difficult cases. Control rates here were variable (0 to 100%), reflecting the challenge of managing drug-resistant epilepsy, but the sample sizes per specific combination are too small for definitive conclusions.

Regarding the mortality rate, a total of 195 cases were included in the analysis, 2 dogs died because of SC, 5 cases died of other diseases and 3 cases were euthanized because of bad quality of life. These data are significantly lower than those reported by Huenerfauth et al. (35), who evaluated dogs with epilepsy for a long-term period. Two dogs died due to uncontrolled SE, the proportion of which is also significantly lower than the research by Fentem et al. (36), as their research also included dogs with structural epilepsy, and the proportion of SE occurring at the time of consultation was also high. This group of data shows that the probability of dogs with IE dying directly from epilepsy itself is only about 1%, the mortality rate is low, and CS basically do not pose a direct threat to life. The direct mortality rate is lower than the one observed by Cagnotti et al. (37), who evaluated dogs experiencing CS and SE. Approximately 1.5% of cases may be euthanized due to the significant distress caused to the dog itself and the pet owner by the occurrence of seizures, SE, or CS. Combined with the information that TTD is approximately 8.2 months, early diagnosis and treatment to control seizures, and reducing the probability of SE and CS, are of great importance for the management of IE. This view is also supported by Heynold et al. (38). In the current retrospective study due to incomplete history, treatment records, and follow-up examinations important pieces of information are missing. Parameters such as clinical manifestations before and after seizures, triggering factors, and the total number of seizures after medication could not be evaluated. Therefore, we call on neurologists to timely and fully improve the diagnostic and treatment information, which is also of great importance for our control and analysis of canine epilepsy.

Regarding regional particularities, the clinical profile of IE in this Chinese cohort appears to be influenced by several factors. First, the predominance of small-breed dogs, such as Poodles and Bichon Frises, aligns with urban pet ownership patterns in China. According to national pet industry data, smaller breeds are preferred in high-density metropolitan areas, which likely explains their high representation in our study compared to Western cohorts. Second, the observed “diagnostic gap” (mean TTD of 8.2 months) indicates a notable interval between the onset of signs and specialist diagnosis. While the availability of MRI hardware increased significantly between 2019 and 2023, the progression to a Tier II diagnosis may be influenced by multifactorial logistical steps, including the time required for owners to monitor initial seizure patterns and the referral process from primary care to specialized centers. Lastly, the low rate of CSF analysis despite high MRI acceptance (75%) suggests that owner decision-making may be influenced by procedure-specific risk perceptions. In our clinical experience, owners may harbor greater apprehension toward invasive diagnostics (e.g., cisterna magnum sampling) compared to non-invasive imaging. However, as structured surveys on owner or practitioner motivations were not part of this retrospective study, these factors remain speculative and warrant further investigation.

This study has several limitations. First, the breed distribution observed in our cohort reflects the population seen at five referral centers rather than a complete national census. While we have incorporated data from the 2024 White Paper on China’s Pet Industry to provide data on canine breed distribution in China for comparison, a genetic predisposition should be interpreted carefully and a high number of two small dog breeds with IE might be more related to a regional representation. Second, the retrospective nature of the study led to incomplete data for certain variables, necessitating the exclusion of some cases; however, a flowchart (Figure 2) has been provided to ensure full transparency of the denominators used for each analysis. Third, while all cases adhered to IVETF guidelines, not all patients underwent MRI/CSF (Tier II) due to owner financial constraints or anesthesia concerns, meaning a subset of the population was diagnosed at Confidence Level I. Despite these limitations, this study provides the largest multicenter dataset to date on canine IE in China.

## 5 Conclusion

In conclusion, this multicenter retrospective study provides the first comprehensive clinical and epidemiological profile of canine idiopathic epilepsy (IE) in China. Our findings demonstrate that the Chinese IE cohort is characterized by a high representation of small-breed dogs and a predominance of generalized seizures. While treatment outcomes with first-line antiseizure medications, specifically Phenobarbital (93.8% efficacy), are highly favorable, the study identifies a notable diagnostic latency, with a mean Time to Diagnosis (TTD) of 8.2 months and approximately 20% of cases exceeding 1 year. These regional findings supplement the global epidemiological map and highlight an objective need for more streamlined diagnostic pathways. By anchoring these results in actual clinical data, this study provides a foundational dataset for future comparative international research and evidence-based management of canine IE in China.

## Data Availability

The original contributions presented in the study are included in the article/supplementary material, further inquiries can be directed to the corresponding authors.
